# Developmental Programming of Nonalcoholic Fatty Liver Disease: The Effect of Early Life Nutrition on Susceptibility and Disease Severity in Later Life

**DOI:** 10.1155/2015/437107

**Published:** 2015-05-18

**Authors:** Minglan Li, Clare M. Reynolds, Stephanie A. Segovia, Clint Gray, Mark H. Vickers

**Affiliations:** Liggins Institute and Gravida: National Centre for Growth and Development, University of Auckland, Auckland 1142, New Zealand

## Abstract

Nonalcoholic fatty liver disease (NAFLD) is fast becoming the most common liver disease globally and parallels rising obesity rates. The developmental origins of health and disease hypothesis have linked alterations in the early life environment to an increased risk of metabolic disorders in later life. Altered early life nutrition, in addition to increasing risk for the development of obesity, type 2 diabetes, and cardiovascular disease in offspring, is now associated with an increased risk for the development of NAFLD. This review summarizes emerging research on the developmental programming of NAFLD by both maternal obesity and undernutrition with a particular focus on the possible mechanisms underlying the development of hepatic dysfunction and potential strategies for intervention.

## 1. Introduction

Nonalcoholic fatty liver disease (NAFLD) is a clinical term which refers to excess fat (>5% weight or volume) deposition in the liver in the absence of excessive alcohol intake. It is rapidly becoming one of the most prevalent liver diseases globally. Population studies utilising ultrasonography and magnetic resonance imaging (MRI) suggest that the prevalence of NAFLD is up to 30% in different countries studied to date including USA, Italy, China, and Japan [1]. Obesity is strongly associated with NAFLD. The incidence of NAFLD in severely obese populations is approximately 74%, and in developed nations 60% of NAFLD patients are obese [2–4]. With obesity rates increasing worldwide, particularly in developing societies undergoing nutritional transition, the prevalence of NAFLD is set to increase markedly in the near future [5, 6].

NAFLD represents a spectrum of pathological changes from isolated hepatic steatosis (fatty liver) without hepatocellular damage to nonalcoholic steatohepatitis (NASH, fatty liver with inflammation) which is the extreme form of the disease characterised by hepatocellular injury and inflammation with or without fibrosis [7]. The natural progression of NAFLD is not fully understood and long-term outcomes are dependent on pathological subtypes. The majority of isolated steatosis has a relatively benign outcome displaying slow progression over many years. However, 10–20% of cases progress to NASH, which is closely linked to hepatic cirrhosis and hepatocellular carcinoma (HCC), and carries a significantly increased mortality risk [8–13].

Although initially considered as a sequential progression from simple steatosis to accompanied inflammation, it is now widely accepted that the pathogenesis of simple steatosis and NASH is likely to progress via different mechanisms [13, 14]. The development of NASH consists of a number of events whereby steatosis and inflammation and cell damage may occur in parallel rather than in strict sequence [13]. The accumulation of fat in hepatocytes can be achieved via four main mechanisms: (1) increased free fatty acid and lipid uptake, (2) increased* de novo* lipogenesis, (3) decreased lipid oxidation, and (4) decreased hepatic very low density lipoprotein (VLDL)-triglyceride secretion, all of which have been reviewed in detail by Fabbrini et al. [15]. On the other hand, in the case of NASH, as proposed by Tilg and Moschen, the evolution of steatosis and inflammation may enhance each other under a number of parallel processes [13]. These processes include, but are not limited to, increased mitochondrial dysfunction, oxidative stress, endoplasmic reticulum (ER) stress, and adipose tissue and gut derived signals such as proinflammatory cytokines, decreased adiponectin, and endotoxin release (“leaky” gut).

**Figure 1:**
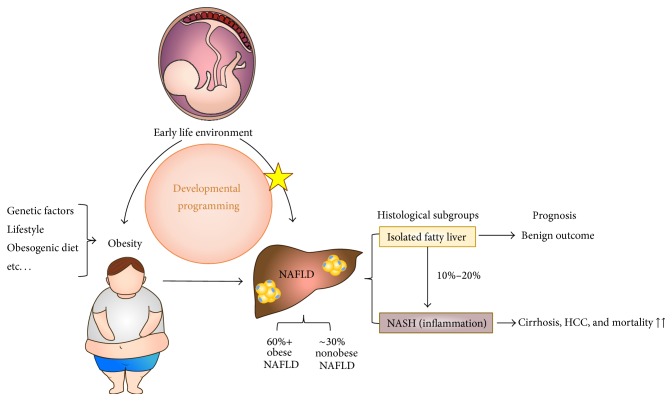
An overview of the development of NAFLD in the context of developmental programming. The developmental programming of NAFLD may occur secondarily to programmed obesity and/or via direct programming effects on the liver. Increased lipid accumulation and inflammation in liver can lead to NASH which is the severe form of NAFLD. NASH is associated with hepatic cirrhosis and HCC and carries a significantly increased mortality risk. NAFLD: nonalcoholic fatty liver disease, NASH: nonalcoholic steatohepatitis, HCC: hepatocellular carcinoma.

In addition to the identified risk factors (including age, obesity, and genetic factors), there is growing evidence that exposure to an unfavourable environment before birth or in early infancy may contribute to an individual's susceptibility and severity of NAFLD through direct effects on the liver and indirect effects via adiposity and metabolic dysfunction. In this review, we focus specifically on the relationships between alterations in the early life developmental environment and potential impact on the occurrence of NAFLD (Figure 1).

## 2. Early Life Nutrition and Metabolic Disorders in Adulthood: Developmental Origins of Health and Disease (DOHaD) Hypothesis

The nutritional environment during preconception, pregnancy, and early life is critical for optimal offspring development and long-term health. Over 20 years ago, Barker and Osmond reported that infant mortality is related to later life ischemic heart disease, suggesting that poor conditions during childhood increase the risk of adult cardiovascular disease [16]. Studies on famine events such as the Dutch Hunger Winter and the Great Chinese Famine have shown that the associations between poor early life nutrition and later life disease are not only limited to cardiovascular disease but also include obesity and the metabolic syndrome [17–19]. Nevertheless, assessing the risk of developmental programming on later health consequences is specific to different contexts and outcomes. The “thrifty phenotype” [20] or predictive adaptive response (PARS) hypotheses have been proposed to explain this phenomenon [21]. These theories argue that poor fetal nutrition leads to metabolic adaptations which act to maximally utilise limited nutrient availability and therefore increase the chances of survival in continued poor conditions after birth. However these adaptations serve to increase the risk for metabolic disorders when exposed to an enriched postnatal nutrient environment. Interestingly, a number of studies on diabetic pregnancies (gestational diabetes and type 2 diabetes) and maternal obesity suggest that excess calorie intake during early life has similar effects on offspring long-term health outcomes [22–25]. Whilst the “thrifty phenotype” or PARS framework may be appropriate in the setting of relative undernutrition, it does not adequately describe the outcomes in offspring observed in the context of maternal obesity. One would argue that offspring of obese mothers would be “matched” to an obesogenic postnatal environment, but offspring of obese mothers display metabolic disorders similar in nature to that observed for maternal undernutrition in the absence of further nutritional insults. This may lie in the observation that excessive maternal caloric intake* per se* may represent a form of fetal malnutrition due to altered placental function and nutrient transport (obesity is commonly associated with micronutrient deficiencies) and thus program the fetus in a way similar to that observed in the setting of direct maternal undernutrition.

The DOHaD hypothesis proposes, from a broad perspective, that alterations in the intrauterine environment can affect the developing fetus in a number of aspects including organogenesis, cell differentiation, and lipid and glucose metabolism, thereby altering risk for development of a range of cardiometabolic disorders in later life [26]. A number of experimental models have provided empirical evidence to support the DOHaD hypothesis including global undernutrition, low protein, and high fat dietary exposures and have provided insights into the physiological and molecular mechanisms linking early life adversity and later disease risk.

## 3. Developmental Programming of NAFLD

### 3.1. Maternal Obesogenic Environment and Offspring NAFLD

In the past few decades there has been mounting evidence to suggest that a maternal obesogenic environment may contribute to offspring obesity and metabolic syndrome [22–25]. However, direct association between maternal obesity and offspring hepatic lipid accumulation in human cohorts was only evidenced in recent years due to the implementation of appropriate diagnostic technologies [27]. It was reported by Modi et al. [28] and Brumbaugh et al. [29] that maternal body mass index (BMI) is directly correlated to neonatal intrahepatocellular lipid content as measured by MRI. In particular, Brumbaugh et al. also showed that the relationship between maternal BMI and neonatal hepatic fat may be independent of neonatal subcutaneous fat leading to speculation that fetal hepatic fat storage may be driven directly by excessive maternal free fatty acid via a pathway distinct from adipose tissue development [29].

A number of experimental animal models using a range of dietary approaches have provided detailed evidence linking a maternal obesogenic environment and the development of NAFLD in offspring. A chronic maternal high fat (HF) diet can lead to a NAFLD phenotype in offspring independent of maternal and offspring obesity [30, 31]. In nonhuman primates (NHP), chronic consumption of a HF diet prior to and during pregnancy, independent of maternal obesity, led to fetal liver steatosis which persisted into the juvenile period [30]. Interestingly, changing the maternal diet to a low fat diet in subsequent pregnancy improved offspring outcome, which highlights that diet during pregnancy has a significant role in the programming of offspring hepatic fat deposition [30]. Similar findings were observed in rodent offspring born to dams chronically consuming a HF diet from preconception to lactation, with a maternal HF diet inducing hepatic steatosis in adult offspring, despite being fed a standard chow diet after weaning [31].

To further investigate whether the severity of NAFLD is influenced by a maternal obesogenic environment, many of the animal models introduced a postweaning HF diet to enhance susceptibility to NAFLD. As expected, when exposed to postweaning HF diet, offspring born to HF diet dams exhibit NASH in early adulthood compared to offspring born to the normal diet dams, which only developed simple steatosis [31–34]. These observations suggest that maternal HF diet increases vulnerability to steatohepatitis rather than simple steatosis in offspring. This is consistent in other dietary models of mixed resource high energy Western and cafeteria diets [35–37]. Kruse et al. demonstrated that offspring exposed to a perinatal HF diet had increased susceptibility to develop NAFLD, despite consuming a normal chow diet for 23 weeks after weaning [38]. This finding emphasises that the pregnancy and lactation period are the critical windows for programming susceptibility to NAFLD. This highlights the irreversibility of such effects in later life, which is consistent with the developmental programming models of other metabolic conditions [39].

In addition to the consumption of a maternal obesogenic diet, preexisting maternal metabolic dysfunction such as insulin resistance also contributes to offspring NAFLD. Thorn et al. compared juvenile NHP born to females chronically exposed to a HF diet with or without development of insulin resistance. Offspring from insulin resistant females, but not insulin sensitive females, developed significant hepatic steatosis despite consuming a healthy diet after weaning and in the absence of obesity [40]. Additionally, an intergenerational study by Li et al. showed that HF feeding through three generations progressively induced severe hepatic steatosis in offspring. Adult offspring from the second generation of HF diet fed animals demonstrated exacerbated NAFLD and increased secretion of the adipokine leptin compared to the previous generation suggesting that programming of NAFLD can accumulate in an intergenerational manner [41].

### 3.2. Early Life Growth Restriction, Undernutrition, and NAFLD

Early life growth restriction discussed here refers to decreased body weight compared to normal birth weight peers, which is commonly observed as a consequence of maternal undernutrition or conditions such as preeclampsia and other forms of placental dysfunction whereby sufficient nutrient supply fails to reach the fetus. Several human studies suggest that early life growth restriction may program liver disease in later life. Fraser et al. reported an association between low birth weight and increased liver enzymes alanine aminotransferase (ALT) and gamma glutamyltransferase (GGT) in a random sample of over 2000 women aged 60–79 years, indicating possible hepatic cellular injury in these subjects [42]. A case control study by Nobili et al. showed an association between paediatric NAFLD and intrauterine growth restriction (IUGR), with low birth weight children demonstrating high prevalence of NASH [43]. However, two other studies suggest that the rapid growth pattern following early growth restriction rather than low birth weight* per se* is strongly associated with the risk of NAFLD. Subjects with accelerated weight gain in the first 3 months of infancy have a significantly higher risk for NAFLD in early adulthood than subjects with slow catch-up growth [44]. An epidemiological study including over 1500 aged participants from the Helsinki Birth Cohort Study showed that childhood body size was negatively associated with NAFLD outcomes after adjustment for adult BMI. Particularly, individuals who were lean in early life and subsequently obese in adulthood had significantly increased risk for NAFLD [45].

In animal models, macronutrient restriction is one of the most commonly used methods to establish offspring growth restriction. Moderate to severe dietary protein restriction during pregnancy and lactation in rats leads to offspring hepatic steatosis in late adulthood without a paralleled increase in adiposity [46, 47]. In sheep, aged lean female offspring born to mothers that received global nutrient restriction in the first half of gestation showed significantly increased hepatic lipid accumulation [48]. Moreover, a study by Yamada et al. showed that hepatic fat deposition occurs in fetuses exposed to maternal undernutrition, as early as embryonic day 20, prior to the development of offspring adiposity [49]. Therefore, it is possible to speculate that growth restriction induced susceptibility to NAFLD is at least partially independent of development of obesity.

In addition to macronutrient restriction, other animal models have shown that factors leading to early growth restriction can also influence liver development. Prenatal hypoxia-induced IUGR increased susceptibility to hepatic steatosis in adulthood [50]. Offspring born to dams subjected to vitamin B12 and folate deficiency have significantly reduced birth weight and hepatic steatosis at weaning [51].

In summary, there is evidence to suggest that obesity, consumption of high fat diets, and undernutrition, during the critical early periods of developmental plasticity, may increase the susceptibility and severity of NAFLD. The programming effect may be partially independent of adiposity. A summary of the related studies is presented in Table 1.

## 4. Potential Mechanisms Involved in the Developmental Programming of NAFLD

### 4.1. Hepatic Lipid Accumulation

The primary feature of NAFLD is accumulation of lipids. Fatty acid accumulation occurs when fatty acid uptake and synthesis exceed hepatocyte oxidative capacity. A human study by Donnelly et al. demonstrated, using stable isotopes, that the dominant source of fat which accumulated in liver originates from serum free fatty acids; this is closely followed by* de novo* lipogenesis [52]. Generally, lipolysis in white adipose tissue (WAT) is the major contributor to serum free fatty acid concentrations [15]. However, this is not the case during fetal life as WAT only starts to develop in the middle of the third trimester in human and NHP and after birth in the rodent [53, 54]. It has been shown in NHP that maternal obesogenic diets induce fetal hepatic steatosis and that fetal and maternal plasma glycerol concentrations are strongly correlated [30]. Fat accumulation in fetal liver may thus originate directly from maternal lipid transfer and represents a “very first hit” of lipotoxicity during early life development. In animal studies, offspring* de novo* lipogenesis can be increased during early adulthood as a result of a maternal obesogenic diet. These various studies have reported increased expression of hepatic transcription factor sterol regulatory element binding protein 1c (SREBP1c) and its coactivators and downstream lipogenic targets: peroxisome proliferator-activated receptors (PPARs), fatty acid synthase (FASN), stearoyl-CoA desaturase-1 (SCD1), and acetyl-CoA carboxylase (ACC1) in adult offspring exposed to obesogenic diets* in utero* [33, 36, 37, 40, 41]. The proposed causes for SREBP1c activation include altered offspring insulin signalling and polyunsaturated fatty acids (PUFAs) metabolism [36, 55–57]. Apart from lipogenesis, the role of hepatic fatty acid *β*-oxidation in the programming of steatosis is not consistent across animal studies. Some find no change in the key enzyme for fatty acid oxidation, carnitine palmitoyltransferase 1 (CPT1) [31, 35], while one study observed persistent decreases in CPT1A gene expression from late gestation to weaning [37]. The synthesis and secretion of VLDL in adult offspring appear to be enhanced by a maternal obesogenic diet [37, 57], which is likely to be a result rather than a cause of hepatic lipid accumulation. Of note, in the maternal obesogenic environment, several genes that are involved in lipid metabolism showed epigenetic modification in adult offspring. Epigenetic modification is considered as a key regulation mechanism in developmental programming [58]. Liver X receptor-*α* (LXR*α*), which is an important mediator for SREBP1c [59], displayed decreased histone methylation after three generations of HF diet consumption in rats, providing a possible explanation for the intergenerational programming effects observed [41]. In a mouse model, alterations in DNA CpG methylation in PPAR*α*, FASN, and insulin-induced gene protein (Insig) were observed in NAFLD offspring with perinatal exposure to Western diet [36]. Overall, these findings suggest that maternal lipid dysregulation and* de novo* lipogenesis have major effects on the developmental programming of offspring hepatic steatosis in the maternal obesogenic setting, with epigenetic modification representing a potential mechanism.

Upon exposure to an undernourished* in utero* environment, increased activation of* de novo* lipogenesis is observed in parallel with the occurrence of fatty liver in rat offspring, with upregulation of hepatic carbohydrate-responsive element-binding protein (ChREBP) and SREBP1c expression at both transcriptional and protein levels [47, 49, 60]. Glucocorticoid exposure is proposed to play a role in the programming of offspring lipogenesis in this setting. It has been demonstrated that maternal protein restriction can lead to reduction of 11 *β*-hydroxysteroid dehydrogenase (11-*β*-HSD) in the placenta and subsequently increases fetal exposure to maternal glucocorticoids [61]. Inhibiting glucocorticoid synthesis reversed the suppressive effect of low protein diet on offspring hepatic SREBP-1c expression [62]. The role of glucocorticoid in programming NAFLD is also supported by Drake et al., who showed that prenatal dexamethasone treatment can increase rat offspring susceptibility to fatty liver without promoting adiposity [63]. However,* in vitro* experiments showed a contrary effect of glucocorticoid on the expression of SREBP1c, suggesting that other factors may be involved in the glucocorticoid effect* in vivo* [62].

### 4.2. Mitochondrial Dysfunction: Oxidative and Endoplasmic Reticulum (ER) Stress

Mitochondria are important organelles which are essential for energy generation and are the primary site for fatty acid *β*-oxidation [64]. The relationship of mitochondrial dysfunction and NAFLD has been reviewed extensively by others [65, 66]. Briefly, mitochondrial fatty acid oxidation increases to adapt to excessive hepatic fat accumulation [67] and in turn leads to a rise in oxidative products—reactive oxygen species (ROS). Most mitochondrial ROS are detoxified to residual molecules through mitochondrial respiratory chain (MRC) activity. However, increased mitochondrial oxidation can progressively induce a vicious cycle including reduction in MRC activity, overproduction of ROS, and damage to mitochondrial DNA. The imbalanced state that favours ROS production over antioxidant defence is defined as oxidative stress. Oxidative stress with excessive mitochondrial ROS has been shown to participate in cell death, inflammation, and fibrosis [68] and therefore may play a significant role in the progression of NASH. In maternal obesogenic animal models, mitochondrial dysfunction and oxidative stress are observed in offspring, reflected by reduced MRC key components—mitochondrial electron transport chain complex (ETC) I, II/III, and IV activity [31], uncoupling MCR activity [69], decreased liver mitochondrial DNA copy number [70], and reduced concentrations of antioxidant enzymes [69, 71]. Nevertheless, it is not completely clear how maternal factors elicit these changes in the next generation. It has been shown by Igosheva et al. that diet induced maternal obesity prior to conception is associated with altered mitochondrial function in mouse oocytes and zygotes [72]. Since mitochondria are maternally inherited, it is possible that the mitochondrial dysfunction in offspring is a combination of inheritance of predisposed maternal mitochondria and exposure to suboptimal early life environment.

The ER is an important organelle for lipid and protein synthesis and export. Disturbance in ER homeostasis (ER stress) has been shown to contribute to both steatosis and the progression to NASH [73, 74]. Emerging evidence suggests that ER stress is associated with* de novo* lipogenesis, mitochondrial dysfunction, oxidative stress, inflammation, and cell death. Details of these interactions have been reviewed elsewhere [75]. In the intergenerational obesogenic diet study, ER stress markers (binding of immunoglobulin protein (BIP), C/EBP homologous protein (CHOP), ER-associated oxidoreductin 1-*α* (ERO1-*α*), and eukaryotic translation initiation factor 2a (eIF2a)) were progressively increased, indicating an intergenerational accumulation of ER stress in these animals [41]. Epigenetic modification on the ERO1-*α* promoter provides a possible explanation for this observation [41].

### 4.3. Proinflammatory Cytokines

NAFLD is linked to obesity and type 2 diabetes, conditions which are associated with chronic low-grade inflammation. Obesity results in altered adipose tissue-derived cytokine and adipokine secretion and progressive infiltration of innate immune cells such as macrophages, which contribute to a state of local and systemic inflammation [76, 77]. This altered inflammatory profile can have peripheral effects on the liver [78], and during pregnancy, can result in enhanced placental and fetal inflammation [79].

It is well established that inflammatory mediators have a critical role in the pathogenesis of NAFLD. In particular, expression of the cytokine tumor necrosis factor-*α* (TNF-*α*) is correlated with the severity of steatohepatitis [80, 81], and reduction of TNF-*α* with metformin improves steatosis in* ob/ob* mice [82]. Free fatty acid accumulation promotes a proinflammatory phenotype through activation of the Toll-like receptor 4 (TLR4) signalling pathway, which culminates in nuclear factor kappa-light-chain-enhancer of activated B cells (NF-*κ*B) activation [83]. Chronic low-level hepatic activation of NF-*κ*B further contributes to hepatic production of TNF*α*, interleukin-1*β* (IL-1*β*), and IL-6 and local and systemic insulin resistance [84]. Furthermore, NAFLD is linked to oxidative stress, ROS production, and generation of toxic lipid peroxides which can damage DNA. Damaged hepatocytes release damage-associated molecular patterns (DAMPs), promoting proinflammatory processes. DAMPs activate the inflammasome, a multiprotein complex responsible for the cleavage of inactive proforms of the proinflammatory cytokines IL-18 and IL-1*β* in the cytoplasm to their bioactive forms, which can then be secreted by the cell [85]. When steatosis occurs, the liver is more susceptible to injury from proinflammatory cytokine stimulation, resulting in progression from NAFLD to NASH. Although the mechanisms underpinning this progression remain unclear, NASH is characterized by hepatocellular degeneration and infiltration of immune and inflammatory cells, which can advance to fibrosis and cirrhosis.

In animal models of maternal obesity, offspring of dams fed a HF diet have significantly reduced natural killer T (NKT) cell populations and upregulated expression of proinflammatory cytokines such as IL-1*β*, IL-6, IL-12, IL-18, and TNF-*α* [31, 32, 34, 86]. Male offspring from dams that consumed a HF diet present clinical features of metabolic syndrome, liver lipid accumulation, and activation of c-Jun N-terminal kinases (JNK) [86]. Pruis et al. demonstrated in mice that exposure to a maternal western diet during pregnancy and/or lactation primed NAFLD in adult male offspring [36]. Early life exposure to a Western-style diet during pregnancy and lactation resulted in hepatomegaly and hepatic cholesterol/triglyceride accumulation, upregulated* de novo* lipid synthesis, and increased expression of inflammatory mediators and macrophage markers including TNF-*α*, transforming growth factor-*β* (TGF-*β*), monocyte chemoattractant protein-1 (MCP-1), and cluster of differentiation 11 (CD11). These changes may be mediated by epigenetic alterations in DNA methylation of PPAR*α*, a transcription factor involved in energy metabolism, hepatic steatosis, and inflammatory processes.

Thorn et al. demonstrated in NHP that* in utero* exposure to HF diet-induced insulin resistance resulted in a programmed increase in hepatic triglycerides and upregulation of hepatic* de novo* lipid synthesis and inflammatory pathways, despite postweaning consumption of a healthy chow diet [40]. Additionally, even though these offspring did not display obesity or insulin resistance, they had both classical and alternatively activated hepatic macrophages and NKT cells, suggesting that maternal insulin resistance programs dysregulation in the juvenile hepatic immune system, which may represent an irreversible “first hit” of NAFLD. Mouralidarane et al. demonstrated in mice that maternal obesity in combination with postweaning consumption of an obesogenic diet induces NAFLD, accompanied by alterations in innate immune function [32]. Kupffer cell (specialized hepatic macrophages), ROS production in response to lipopolysaccharide, and hepatic inflammatory cytokines IL-12 and IL-18 were increased in maternal obesity offspring compared to normal offspring when both exposed to a postweaning obesogenic diet. These findings suggest that maternal obesity predisposes offspring to development of NAFLD through alterations in the innate immune system, which is exacerbated by postnatal consumption of a hypercalorific diet. However, as obesity, insulin resistance, and NAFLD commonly occur together in humans and are all linked with inflammatory processes, disentangling the specific pathways involved in the developmental programming of NAFLD remains a challenge.

**Figure 2:**
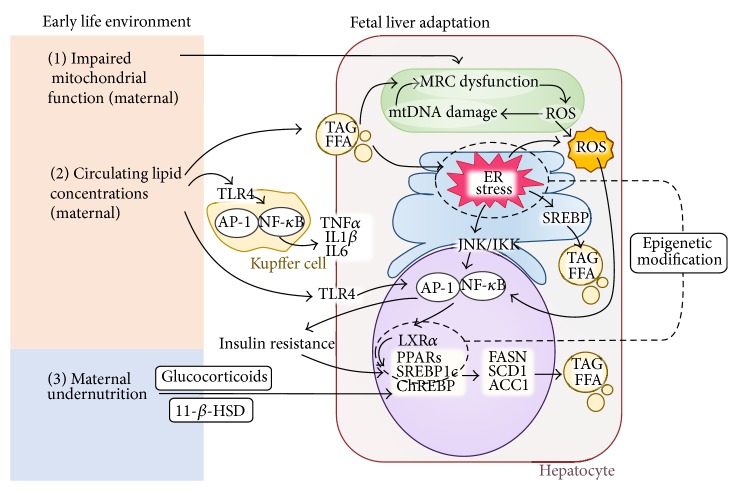
Potential mechanisms underlying the developmental programming of NAFLD. (1) Maternal obesity and high fat diet induced mitochondrial dysfunction may be programmed in the fetus; (2) maternal circulating lipids are shuttled to the fetal liver contributing to mitochondrial oxidative stress; this is characterised by reduced MRC activity, overproduction of ROS, and mitochondrial DNA damage. Increased concentrations of TAG and FFA contribute to ER stress which can induce additional oxidative stress, increase* de novo* lipogenesis, and activate inflammatory responses via JNK/NF-*κ*B pathway. Lipid toxicity can active inflammation via TLR4 signalling pathway in both Kupffer cells and hepatocytes, where the former is a major source of proinflammatory cytokines including TNF*α*, IL1*β*, and IL6. Chronic low-level hepatic NF-*κ*B activation further contributes to local and systemic insulin resistance, which in turn influences* de novo* lipogenesis. (3) Maternal undernutrition can reduce 11-*β*-hydroxysteroid dehydrogenase (11-*β*-HSD) in the placenta and therefore increase fetal exposure to maternal glucocorticoids. Increased glucocorticoids can lead to fetal* de novo* lipogenesis. Markers that indicate ER stress and* de novo* lipogenesis can be modified by early life epigenetic mechanism which may represent a path for intergenerational transmission of disease risk. MRC: mitochondrial respiratory chain; ROS: reactive oxygen species; TAG: triglyceride; FFA: free fatty acid; ER: endoplasmic reticulum; SREBP: sterol regulatory element binding protein; JNK: c-Jun N-terminal kinase; IKK: I*κ*B kinase; NF-*κ*B: nuclear factor kappaB; AP-1: activator protein 1; TLR4: Toll-like receptor 4; LXR*α*: Liver X receptor-*α*; PPARs peroxisome proliferator-activated receptors; ChREBP: carbohydrate-responsive element-binding protein; FASN: fatty acid synthase; SCD1: stearoyl-CoA desaturase-1; ACC1: acetyl-CoA carboxylase; 11-*β*-HSD: 11-*β*-hydroxysteroid dehydrogenase.

Overall, the mechanisms involved in the developmental programming of NAFLD are multifactorial. At a molecular level,* de novo* lipogenesis, primed mitochondrial and ER dysfunction, and the activation of inflammatory response are the main pathways that are most likely to have long lasting adaptations under different early life environments. Potential mechanisms that contribute to the developmental programming of NAFLD are summarised in Figure 2.

## 5. Sexual Dimorphism in Programming NAFLD

Although initially thought to be more common in females [7], recent evidence shows that the prevalence of NAFLD is higher in males [87–89]. In particular, paediatric NAFLD is more prevalent in boys, with a male to female ratio of 2.5 : 1 [90, 91]. Although the majority of animal studies only examine male offspring due to the potential confounds of estrus, there is some evidence suggesting that female offspring are likely to be moderately protected from NAFLD in the maternal obesogenic environment. Bayol et al. reported that a maternal junk food diet promotes exacerbated steatosis and hepatocyte ballooning in both male and female offspring. However, increased expression of genes associated with* de novo* lipogenesis and lipid oxidation were only observed in males [35]. Strakovsky et al. found that feeding a HF diet to an obesity resistant strain of rats during pregnancy led to a significant increase in hepatic triglycerides in male neonates but not females with a sex-specific change in the antioxidative system [92]. In another maternal HF diet model, HF feeding during early life programmed hepatic steatosis and insulin resistance in male offspring chronically exposed to HF diet in adult life, whereas female offspring were protected from the NAFLD phenotype [70]. One of the potential explanations for this disparity is the liver-protective role of estrogens, as well as the potential role of androgens in aggravating NASH [91, 93]. Nevertheless, sexual dimorphism is frequently observed in developmental programming models, with molecular and phenotypic outcomes of adverse* in utero* conditions often more prominent in male offspring [94].

## 6. Potential Intervention Strategies during Early Life

Work by Godfrey et al. highlighted that the earlier the intervention during the life course the greater the impact on later life health and well-being of offspring [95]. Intervention strategies to ameliorate the developmental programming of NAFLD need to be introduced in early life during critical windows of developmental plasticity to elicit the most effective benefits. Animal models indicate that programmed effects are highly irreversible after weaning; long-term consumption of a normal chow diet after weaning may not be effective in normalising offspring susceptibility to NAFLD induced by maternal HF diet [35, 38].

Evidence suggests that breastfeeding may confer some protection against the development of NAFLD in humans. A study of 191 children with NAFLD showed that early breast feeding may have a protective effect on the progression to NASH and liver fibrosis independent of the present or neonatal characteristics of the children [96]. It has been shown in other studies that longer duration of breastfeeding can decrease the risk of offspring becoming overweight in later life [97]. Breast milk is a rich source of long-chain PUFAs such as eicosapentaenoic acid (EPA) and docosahexaenoic acid (DHA) [98]. It has been reported that PUFAs can suppress* de novo* lipogenesis via inhibition of SREBP1c [99]. Moreover, DHA can act as a PPAR-agonist reducing experimental liver fibrogenesis in mice [100], hence having a preventive effect on NASH. Breast milk also contains numerous peptides such as insulin and leptin (which are not present in infant formula) that are bioactive and may influence infant growth and body composition [101]. In particular, leptin administration during the neonatal period reverses developmental programming of metabolic disorders in the rat [102]. Although these interventions have not been tested directly in the setting of NAFLD, these factors have the potential to protect against the progression of hepatic steatosis.

Several dietary supplements have been investigated in animal models as intervention strategies to combat adverse developmental programming effects. Fish oil is naturally enriched in PUFAs and has shown antiobesity effects in animal models [103]. Bringhenti et al. reported that introducing fish oil to a postweaning diet can reverse maternal low protein diet induced hepatic steatosis in offspring [104]. This effect is likely achieved via a reduction in* de novo* lipogenesis and enhanced lipid oxidation [104]. The plant extract resveratrol, which is a naturally occurring compound of various fruits such as red grapes, is known to have multiple chemoprotective properties including antioxidant and anti-inflammatory effects [105]. Franco et al. reported that, although given in adulthood, resveratrol reversed early weaning induced adult offspring liver steatosis and dyslipidemia [106]. This may be due to the beneficial effect of resveratrol on mitochondrial oxidative stress [106, 107]. The progress from NAFLD to NASH is critically regulated by proinflammatory cytokines as discussed previously. Taurine is a sulfonic amino acid with anti-inflammatory properties [108]. A recent study by our group suggested that taurine supplementation during pregnancy and lactation may ameliorate an adverse proinflammatory hepatic profile observed in offspring following a maternal obesogenic diet [109]. This may potentially reduce the susceptibility to NASH by moderating inflammatory responses upon exposure to insults. Even though these supplementations look promising, further thorough experiments regarding safety profiles are required before implementation as a therapeutic option.

## 7. Summary

The development of NAFLD is a multifactorial process. In addition to obesity, age, genetic factors, and lifestyle, suboptimal early life nutrition including a maternal obesogenic environment or undernutrition may increase the susceptibility, age of onset, and severity of the disease. The influence of early life nutrition on the development of NAFLD is likely in part independent of adiposity. Animal models representing different maternal nutritional insults provide in-depth views on the mechanisms relating to hepatic lipid accumulation and the progression to liver inflammation. Particularly, maternal lipid dysregulation and later life* de novo* lipogenesis are the major contributors for offspring hepatic steatosis in the maternal obesogenic setting, while in a growth restricted environment, glucocorticoid alteration is proposed to play an important role in the development of offspring fatty liver. Furthermore, mitochondrial dysfunction, oxidative stress, ER stress, and inflammatory responses are all involved in the progression of the disease in the setting of developmental programming. While breastfeeding shows a possible protective effect, dietary supplements with anti-inflammatory and antioxidant capacity may also have the potential to reduce further increases in NAFLD, partly attributed to poor* in utero* and early life nutritional programming.

## Figures and Tables

**Table 1:** A summary of human and animal studies related to the developmental programming of NAFLD.

Early life insults	Species	Offspring NAFLD	Influence on offspring adiposity	References
Maternal obesogenic environment				
Increased maternal BMI	Human	Increased neonatal hepatic lipid content	Independent of neonatal subcutaneous fat	[28, 29]
Maternal chronic HF diet consumption	NHP	Fetal hepatic steatosis persisting to juvenile age	No increase in body weight or body fat	[30]
Maternal chronic HF diet consumption	Mouse	Hepatic steatosis in offspring with postweaning chow diet; NASH in offspring with postweaning HF diet	Increase in fat accumulation, with highest increase in offspring with postweaning HF diet	[31]
Maternal HF diet	Mouse; rat	NASH in offspring with postweaning HF diet [33, 38, 57]; hepatic steatosis in offspring with postweaning chow diet [86]	Increased adiposity	[33, 38, 57, 86]
Maternal obesogenic diet (mixed source)	Mouse; rat	NASH in offspring with postweaning obesogenic diet [32, 34–37]; increased hepatic lipid accumulation at early age with postnatal chow diet [69]	Increased body weight/adiposity [32, 34–37]; no changes in body weight or adiposity [69]	[32, 34–37, 69]
HF diet induced maternal insulin resistance	NHP	NAFLD in offspring with postweaning chow diet	No obesity present	[40]
Intergenerational HF diet	Mice	Progressive exacerbation of NAFLD	Progressively increased adiposity	[41]
Growth restriction/maternal UN				
Low birth weight	Human	Increased plasma ALT and GCT at 60–79 years	Adjusted for waist-to-hip ratio	[42]
Small for gestational age	Human	Independently associated with NAFLD	After correction for BMI	[43]
Accelerated weight gain in the first 3 months of infancy	Human	Increased risk for NAFLD in early adulthood	After correction for adult weight *Z* score	[44]
Lean in early life and subsequently obese	Human	Increased risk for NAFLD	Adjusted for adult BMI	[45]
Maternal low protein diet	Rat	Hepatic steatosis	Without a parallel increase in adiposity	[46, 47]
Maternal global nutrient restriction	Sheep	Hepatic lipid accumulation in aged offspring	Offspring are lean	[48]
Maternal undernutrition	Rat	Fetal hepatic fat deposition at embryonic day 20	Prior to the development of offspring adiposity	[49]
Prenatal hypoxia induced IUGR	Rat	Hepatic steatosis in offspring with hypoxia challenge at age of 6 months	No change in body weight	[50]
Vitamin B12 and folate deficiency induced IUGR	Rat	Hepatic steatosis at weaning	Significantly decreased body weight	[51]
